# Combined C-reactive protein, procalcitonin, and microalbuminuria panel for contrast-induced nephropathy risk stratification after percutaneous coronary intervention

**DOI:** 10.3389/fcvm.2026.1899776

**Published:** 2026-09-09

**Authors:** XueChao Yuan, ZeXiang Xu, Xin Guo

**Affiliations:** Department of Cardiovascular Medicine, Cangzhou Central Hospital, Cangzhou, China

**Keywords:** biomarker panel, cancer cardiology, cardio-oncology, contrast-induced nephropathy, C-reactive protein, microalbuminuria, procalcitonin

## Abstract

**Background:**

Cancer patients with cardiovascular comorbidities requiring contrast-based cardiac procedures face substantially amplified risk for contrast-induced nephropathy (CIN), driven by anticancer therapy-related systemic inflammation, nephrotoxicity, and endothelial dysfunction. Validated biomarker-based risk stratification tools that capture these distinct pathophysiologic mechanisms are urgently needed in cancer cardiology practice. This study evaluated the combined predictive value of C-reactive protein (CRP), procalcitonin (PCT), and microalbuminuria (MAU) for CIN development following percutaneous coronary intervention (PCI), with direct implications for cardio-oncology risk stratification.

**Methods:**

We retrospectively analyzed 486 consecutive patients undergoing elective or urgent PCI between March 2021 and November 2024. Baseline CRP, PCT, and MAU were measured within 24 h pre-procedure. CIN was defined as serum creatinine increase ≥0.5 mg/dL or ≥25% from baseline within 48–72 h post-contrast.

**Results:**

CIN developed in 92 patients (18.9%). All three biomarkers independently predicted CIN in multivariable analysis: CRP >10 mg/L (OR = 3.15, *p* < 0.001), PCT >0.05 ng/mL (OR = 2.87, *p* < 0.001), and MAU ≥30 mg/24 h (OR = 3.42, *p* < 0.001). The combined three-biomarker panel demonstrated superior discrimination (AUC = 0.856, 95% CI: 0.818–0.894) compared to individual markers (AUC 0.682–0.728) and the Mehran score (AUC = 0.714). An additive biomarker score (0–3) stratified CIN risk from 5.8% (score 0) to 50.0% (score 3), representing an approximately8.6-fold gradient.

**Conclusion:**

Combined pre-procedural assessment of CRP, PCT, and MAU provided excellent discriminatory ability (AUC=0.856) for CIN prediction, substantially superior to individual biomarkers and traditional clinical risk factors. These complementary markers—capturing systemic inflammation, acute inflammatory stress, and baseline renal endothelial integrity, respectively—offer a clinically practical framework for biomarker-guided risk stratification in a general PCI population. As patients on active systemic anticancer therapy were excluded and patients with a cancer history comprised only a minority of the cohort, the potential extension of this panel to cardio-oncology practice is proposed as a mechanistically motivated hypothesis that requires dedicated validation rather than as a demonstrated application.

## 1 Introduction

The intersection of cancer biology and cardiovascular pathophysiology represents one of the most urgent and rapidly evolving frontiers in contemporary medicine. Anticancer therapies—encompassing cytotoxic chemotherapy, targeted molecular agents, immune checkpoint inhibitors (ICIs), adoptive cell therapies, and ionizing radiation—have dramatically improved cancer survival outcomes, with over 18 million cancer survivors currently living in the United States alone. However, these treatments impose substantial and often irreversible cardiovascular burden, contributing to hypertension, cardiomyopathy, myocarditis, arrhythmias, vascular toxicity, and complex cardiorenal complications that significantly impair long-term survival and quality of life. The specialized field of cardio-oncology has emerged to address these overlapping and mutually amplifying pathologies, advocating for integrated biomarker-based surveillance, dynamic risk stratification, and individualized preventive strategies across the cancer treatment continuum (1–5).

A critical and underrecognized challenge in cancer cardiology is contrast-induced nephropathy (CIN) complicating cardiac catheterization and contrast-based procedures in cancer patients with cardiovascular comorbidities. Anthracycline-induced cardiomyopathy, radiation-associated coronary artery disease, and vascular endothelial injury from antiangiogenic agents create complex cardiovascular phenotypes necessitating coronary angiography and percutaneous coronary intervention (PCI). In this population, CIN risk is substantially amplified through multiple cancer therapy-related mechanisms: nephrotoxic chemotherapy (cisplatin, carboplatin, ifosfamide, methotrexate) progressively reduces renal reserve; checkpoint inhibitor-associated immune nephritis impairs glomerular integrity; antiangiogenic agents targeting vascular endothelial growth factor (VEGF) disrupt renal microvascular autoregulation; systemic inflammatory states from malignancy and immunotherapy elevate baseline vascular vulnerability; and treatment-related endothelial dysfunction compounds the direct tubular nephrotoxicity of iodinated contrast media (6–9). CIN in cancer patients carries particularly grave prognostic implications, potentially forcing postponement or dose reduction of life-sustaining anticancer treatments, accelerating chronic kidney disease progression, and independently worsening oncological outcomes (10).

CIN is defined as an acute decline in renal function following intravascular contrast media administration, typically manifesting as serum creatinine increase ≥0.5 mg/dL or ≥25% from baseline within 48–72 h, with reported incidences of 7%–25% in general percutaneous coronary intervention cohorts and potentially exceeding 30% in cancer patients with cumulative nephrotoxic treatment exposures (11–13). The pathophysiology is multifactorial, involving direct tubular toxicity, medullary hypoxia from altered hemodynamics, oxidative stress with reactive oxygen species generation, and endothelial dysfunction—mechanisms that substantially overlap with those activated by anthracyclines, alkylating agents, and immune checkpoint inhibitors (14–16). Traditional risk prediction tools, most notably the Mehran score, demonstrate only modest discriminatory performance (AUC 0.65–0.72) in general populations and incorporate exclusively clinical and procedural variables without dynamic biomarkers capturing the inflammatory and renal endothelial states that are particularly relevant—and frequently disrupted—in cancer cardiology patients (17, 18).

Three biomarkers offer particular mechanistic and clinical relevance to CIN risk stratification in cancer cardiology contexts. C-reactive protein (CRP), an acute-phase reactant synthesized by hepatocytes in response to interleukin-6, serves as an integrative marker of systemic inflammation—a state commonly amplified by malignancy itself, cytokine release from immunotherapy, and checkpoint inhibitor-associated immune activation. Elevated CRP reflects endothelial dysfunction and pro-inflammatory vascular states that potentiate contrast nephrotoxicity through impaired nitric oxide bioavailability, complement activation, and heightened oxidative stress (19, 20). Procalcitonin (PCT), a calcitonin precursor released during acute inflammatory stress, exhibits rapid kinetics potentially capturing transient inflammatory cascades—including cytokine release syndrome from CAR-T cell therapy and bispecific antibodies, immune-related adverse events from checkpoint inhibitors, and subclinical immunotherapy-induced organ toxicity—that predispose the renal microvasculature to ischemic injury (21, 22). Microalbuminuria (MAU), defined as urinary albumin excretion 30–300 mg/24 h, represents early glomerular and endothelial dysfunction reflecting the cumulative renal impact of nephrotoxic chemotherapy, antiangiogenic agents, radiation nephropathy, and treatment-related hypertension. MAU thus serves as an integrative biomarker of reduced nephron reserve and heightened susceptibility to additional injurious stimuli—a profile characteristic of cancer patients with cumulative anticancer treatment exposures (23, 24).

No study has examined the combined predictive value of these three mechanistically complementary biomarkers—reflecting systemic inflammation (CRP), acute inflammatory stress (PCT), and baseline renal endothelial integrity (MAU)—for CIN risk stratification in a framework directly applicable to cardio-oncology practice. We hypothesized that their integration would provide superior discriminatory ability compared to traditional clinical risk factors alone (25). The primary objective of this study was to evaluate the combined predictive value of pre-procedural CRP, PCT, and MAU for CIN development after PCI. Secondary objectives included determining optimal biomarker cut-offs, comparing discriminatory ability against individual markers and the Mehran score, developing an integrated additive biomarker risk score, and delineating clinical implications for cancer cardiology practice including risk-adapted preventive strategies and post-procedural monitoring protocols.

## 2 Materials and methods

### 2.1 Study design

This retrospective observational cohort study was conducted at Cangzhou Central Hospital, Cangzhou, China, between March 2021 and November 2024. The study was designed according to STROBE guidelines, approved by the Institutional Review Board (approval 2023-129-02(z)). Owing to the retrospective design, the requirement for written informed consent was waived by the Institutional Review Board, and patient data were anonymized prior to analysis. The biomarker-based risk stratification framework investigated is also relevant to cancer cardiology clinical practice, where validated pre-procedural predictive tools reflecting inflammatory and renal endothelial dimensions of cardiovascular risk are lacking; however, the present cohort was not enriched for patients receiving active anticancer therapy, and this application is therefore explored as a hypothesis rather than a validated indication.

### 2.2 Patient population

Consecutive patients who underwent PCI at Cangzhou Central Hospital between March 2021 and November 2024 were retrospectively identified from the hospital information system.

Inclusion criteria: (1) age ≥18 years; (2) angiographically confirmed coronary artery disease (≥50% stenosis in a major epicardial vessel); (3) PCI performed (elective, urgent, or emergency) at Cangzhou Central Hospital, March 2021–November 2024; (4) complete pre-procedural CRP, PCT, and 24-hour urine albumin data available in the medical record; (5) serum creatinine measurements available at 48 and 72 h post-procedure.

Exclusion criteria: (1) severe chronic kidney disease (eGFR <30 mL/min/1.73 m^2^ or dialysis-dependent at baseline); (2) contrast media administration within 7 days prior to the index procedure; (3) acute kidney injury at presentation; (4) active infection or sepsis; (5) recent surgery or trauma within 14 days; (6) malignancy under active systemic anticancer treatment; (7) pregnancy; (8) documented contrast allergy; (9) missing baseline biomarker or post-procedural creatinine follow-up data.

### 2.3 Pre-procedural evaluation

All patients underwent comprehensive evaluation within 24 h pre-procedure including: detailed medical history (including prior cancer diagnoses and anticancer treatment history), physical examination, laboratory testing (complete blood count, comprehensive metabolic panel with serum creatinine, fasting glucose or HbA1c, lipid panel, NT-proBNP), baseline eGFR calculation using the CKD-EPI equation, and echocardiography to assess left ventricular ejection fraction (LVEF) using Simpson's biplane method.

### 2.4 Biomarker measurement

Blood samples for CRP and PCT were collected within 24 h pre-procedure. High-sensitivity CRP was measured using immunoturbidimetric assay (Roche Diagnostics, range 0.15–350 mg/L, CV <5%). PCT was measured using electrochemiluminescence immunoassay (Roche Cobas e601, detection limit 0.02 ng/mL, CV <7%). Because mild PCT elevations may reflect occult infection, patients with clinical or laboratory evidence of active infection (fever, leukocytosis, positive cultures, or a documented infectious diagnosis) were excluded at screening, and the >0.05 ng/mL threshold was therefore applied only to patients without overt infection. For microalbuminuria assessment, 24 h urine collection was initiated at hospital admission. For elective cases the collection was completed before the procedure, whereas for urgent/acute coronary syndrome presentations collection was initiated immediately on admission and continued peri-procedurally, with the timed sample used for albumin quantification. Collection completeness was verified using expected 24 h urinary creatinine excretion (15–20 mg/kg for women and 20–25 mg/kg for men); samples outside this range were repeated, and 22 collections (4.5%) were initially inadequate and recollected. Urinary albumin concentration was measured using immunoturbidimetric assay. MAU was defined as albumin excretion 30–300 mg per 24 h. All biomarker measurements were performed by laboratory personnel blinded to clinical data and outcomes.

### 2.5 PCI procedure

All procedures were performed by experienced interventional cardiologists. Vascular access: radial artery (preferred) or femoral artery. Low-osmolar non-ionic contrast media (iopamidol 370 mg I/mL or iohexol 350 mg I/mL) were used exclusively; no iso-osmolar agent was administered. Iopamidol was used in 268 patients (55.1%) and iohexol in 218 patients (44.9%), with comparable distribution between the CIN and non-CIN groups (iopamidol: 50/92 [54.3%] vs. 218/394 [55.3%], *p* = 0.86). Contrast volume was recorded and minimized per protocol. The contrast volume-to-creatinine clearance ratio (CV/CrCl) was calculated using the Cockcroft-Gault equation.

### 2.6 Hydration protocol

Intravenous hydration with 0.9% normal saline was administered per standardized protocol. For elective procedures: 1.0 mL/kg/hour starting 6–12 h pre-procedure and continuing 6–12 h post-procedure, adjusted for LVEF and volume status. For urgent/emergency procedures: 1.0–1.5 mL/kg/hour initiated immediately. Patients with LVEF <40% received 0.5 mL/kg/hour. Diuretics were held 24 h pre-procedure when feasible.

### 2.7 CIN definition and follow-up

Serum creatinine was measured at baseline and at 24, 48, and 72 h post-contrast. CIN was defined as increase in serum creatinine ≥0.5 mg/dL (44.2 μmol/L) or ≥25% increase from baseline, occurring within 48–72 h post-contrast, not attributable to alternative causes (12, 17). This classical definition was selected deliberately to preserve comparability with the large body of prior PCI-based CIN literature and with the Mehran risk score against which our model is benchmarked; we acknowledge that contemporary KDIGO and AKIN contrast-associated acute kidney injury criteria apply lower creatinine thresholds, and validation of the panel against these criteria is warranted in future work. All follow-up data, including post-procedural serum creatinine measurements at 24, 48, and 72 h, length of stay, in-hospital mortality, and chronic kidney disease status at 3-month follow-up, were ascertained from existing medical records and routine outpatient documentation; no patients were actively contacted for research follow-up. CIN events were adjudicated by two independent nephrologists blinded to biomarker data. Secondary outcomes included: severe CIN requiring dialysis, peak creatinine rise, length of stay, in-hospital mortality, and chronic kidney disease at 3-month follow-up.

### 2.8 Statistical analysis

Sample size calculations estimated 450 patients required for 80% power to detect an AUC of 0.75 or higher for the combined biomarker model. Continuous variables are expressed as mean ± SD or median (IQR). Categorical variables are expressed as frequencies and percentages. Group comparisons used Student's t-test or Mann–Whitney U test for continuous, and chi-squared or Fisher's exact test for categorical variables. Optimal biomarker cut-offs were determined using ROC curve analysis maximizing the Youden index. Logistic regression identified independent CIN predictors through univariable analysis followed by multivariable backward stepwise selection. Model discrimination was assessed using AUC with 95% CI. Model calibration was evaluated using the Hosmer-Lemeshow test. Internal validation used bootstrap resampling (1,000 iterations). Net reclassification improvement (NRI) and integrated discrimination improvement (IDI) assessed the incremental predictive value of the biomarker panel over the Mehran score. Two-sided *p* < 0.05 was considered statistically significant. Statistical analyses were performed using SPSS 27.0 and R 4.3.1.

## 3 Results

### 3.1 Study population and patient flow

Between March 2021 and November 2024, 538 patients were screened; 52 were excluded (28 had missing baseline biomarker or follow-up data, 12 had severe CKD, 6 had received recent contrast, 4 had active infection, and 2 had incomplete 24-hour urine collection). The final analytic cohort comprised 486 patients. Mean age was 65.8 ± 9.2 years; 153 (31.5%) were female. Clinical presentation: stable angina 198 (40.7%), unstable angina 162 (33.3%), NSTEMI 85 (17.5%), STEMI 41 (8.4%). Mean baseline eGFR was 78.5 ± 22.3 mL/min/1.73 m^2^. Comorbidities included hypertension in 352 (72.4%), diabetes in 167 (34.4%), current smoking in 142 (29.2%), prior myocardial infarction in 98 (20.2%), heart failure in 56 (11.5%), and peripheral arterial disease in 38 (7.8%). A documented history of malignancy was present in 58 patients (11.9%), all of whom had stable disease and none of whom were receiving active systemic anticancer therapy at the time of the procedure.

### 3.2 Procedural characteristics

PCI was technically successful in all 486 patients. Radial access was used in 382 (78.6%), femoral in 104 (21.4%). Single-vessel PCI was performed in 318 (65.4%); multi-vessel PCI in 168 (34.6%). Median stents implanted: 2 (IQR: 1–3). Median contrast volume: 168 mL (IQR: 120–225). High contrast volume (>200 mL) was used in 182 patients (37.4%). Median CV/CrCl ratio: 2.1 (IQR: 1.5–3.2). Procedural complications occurred in 18 patients (3.7%): no-reflow phenomenon (*n* = 8), coronary dissection (*n* = 5), perforation (*n* = 2), stroke (*n* = 1), major bleeding (*n* = 2). No procedural mortality was recorded.

### 3.3 Baseline biomarker levels

Median baseline CRP was 5.8 mg/L (IQR: 2.4–12.5); elevated CRP (>10 mg/L) was present in 168 patients (34.6%). Median baseline PCT was 0.04 ng/mL (IQR: 0.02–0.08); elevated PCT (>0.05 ng/mL) was present in 142 patients (29.2%). Microalbuminuria (30–300 mg/24 h) was detected in198 patients (40.7%), including 156 with microalbuminuria (30–300 mg/24 h) and 42 with overt proteinuria (>300 mg/24 h), all classified as MAU-positive (albumin excretion ≥30 mg/24 h); normal albumin excretion (<30 mg/24 h) was present in 288 patients (59.3%). Biomarker correlations were statistically significant: CRP-PCT (r = 0.58, *p* < 0.001), CRP-MAU (r = 0.48, *p* < 0.001), PCT-MAU (r = 0.52, *p* < 0.001), reflecting shared inflammatory and endothelial pathways.

### 3.4 CIN incidence and baseline characteristics

CIN developed in 92 patients (18.9%, 95% CI: 15.5–22.7%). Mean peak creatinine increase was 0.78 ± 0.32 mg/dL at a median of 48 h (IQR: 36–60) post-contrast. Seven patients (1.4%) required temporary dialysis; four subsequently recovered renal function, while three remained dialysis-dependent at 90 days. CIN patients were significantly older (68.5 ± 8.8 vs. 65.1 ± 9.2 years, *p* < 0.001), had higher diabetes prevalence (52.2% vs. 30.2%, *p* < 0.001), lower baseline eGFR (64.2 ± 20.8 vs. 82.1 ± 21.2 mL/min/1.73 m^2^, *p* < 0.001), received higher contrast volumes (205 ± 68 vs. 152 ± 58 mL, *p* < 0.001), and had markedly higher biomarker levels across all three markers. Table 1 presents detailed baseline characteristics stratified by CIN status. Periprocedural statin therapy (overall 81.9%, 398/486) and N-acetylcysteine administration (overall 26.5%, 129/486) were comparably distributed between the CIN and non-CIN groups (statin 79.3% vs. 82.5%, *p* = 0.47; N-acetylcysteine 25.0% vs. 26.9%, *p* = 0.71), and neither was a significant predictor of CIN.

**Table 1:** Baseline characteristics stratified by CIN Status.

Characteristic	No CIN (*n* = 394)	CIN (*n* = 92)	*P*-value
Age (years)	65.1 ± 9.2	68.5 ± 8.8	<0.001
Female sex	122 (31.0%)	31 (33.7%)	0.609
BMI (kg/m^2^)	25.8 ± 3.4	26.2 ± 3.7	0.308
Hypertension	281 (71.3%)	71 (77.2%)	0.261
Diabetes mellitus	119 (30.2%)	48 (52.2%)	<0.001
Current smoking	116 (29.4%)	26 (28.3%)	0.832
Prior MI	76 (19.3%)	22 (23.9%)	0.321
Heart failure	38 (9.6%)	18 (19.6%)	0.007
Peripheral arterial disease	27 (6.9%)	11 (12.0%)	0.111
Stable angina	165 (41.9%)	33 (35.9%)	0.309
Unstable angina	128 (32.5%)	34 (37.0%)	0.429
NSTEMI	66 (16.8%)	19 (20.7%)	0.392
STEMI	35 (8.9%)	6 (6.5%)	0.473
Baseline Cr (mg/dL)	0.98 ± 0.26	1.18 ± 0.34	<0.001
Baseline eGFR (mL/min/1.73 m^2^)	82.1 ± 21.2	64.2 ± 20.8	<0.001
LVEF (%)	56.8 ± 10.2	52.4 ± 12.5	0.001
CRP (mg/L) [median (IQR)]	4.8 (2.2–9.5)	15.2 (8.8–28.4)	<0.001
CRP >10 mg/L	108 (27.4%)	60 (65.2%)	<0.001
PCT (ng/mL) [median (IQR)]	0.03 (0.02–0.06)	0.08 (0.05–0.15)	<0.001
PCT >0.05 ng/mL	92 (23.4%)	50 (54.3%)	<0.001
MAU ≥30 mg/24 h	105 (26.6%)	51 (55.4%)	<0.001
Contrast volume (mL)	152 ± 58	205 ± 68	<0.001
Contrast volume >200 mL	122 (31.0%)	60 (65.2%)	<0.001
CV/CrCl ratio	2.0 ± 1.2	3.2 ± 1.5	<0.001
Statin therapy	325 (82.5%)	73 (79.3%)	0.47
N-acetylcysteine (periprocedural)	106 (26.9%)	23 (25.0%)	0.71

Values are mean ± SD, median (IQR), or *n* (%).

BMI, body mass index; MI, myocardial infarction; NSTEMI, non-ST-elevation MI; STEMI, ST-elevation MI; Cr, creatinine; eGFR, estimated glomerular filtration rate; LVEF, left ventricular ejection fraction; CRP, C-reactive protein; PCT, procalcitonin; MAU, microalbuminuria; CV/CrCl, contrast volume-to-creatinine clearance ratio.

### 3.5 Individual biomarker performance

**Figure 1:**
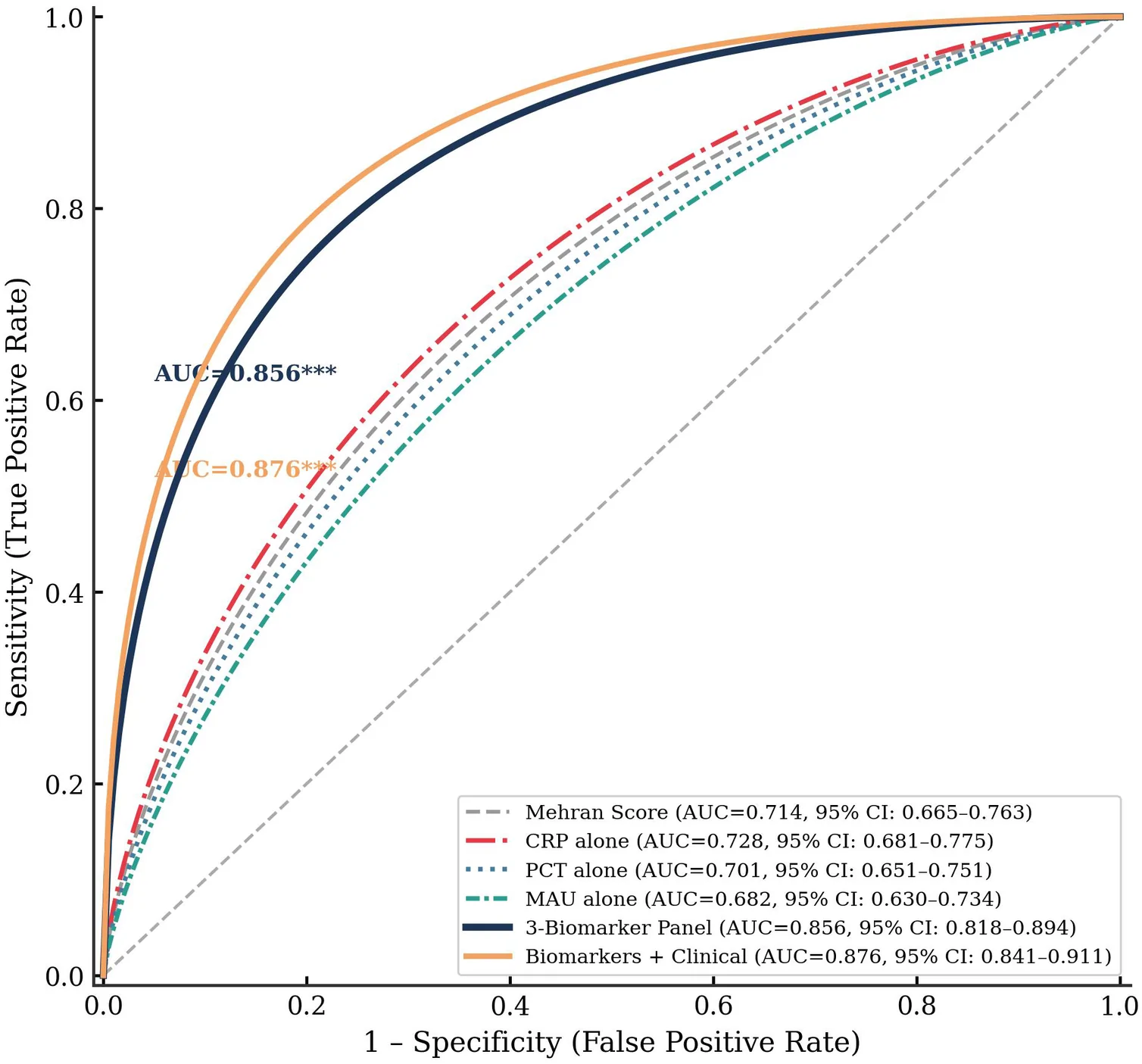
ROC curves for individual biomarkers and combined models predicting contrast-induced nephropathy. The combined three-biomarker panel (AUC = 0.856, 95% CI: 0.818–0.894) demonstrated significantly superior discrimination compared to CRP alone (AUC = 0.728, *p* < 0.001), PCT alone (AUC = 0.701, *p* < 0.001), MAU alone (AUC = 0.682, *p* < 0.001), and the Mehran score (AUC = 0.714, *p* < 0.001). The integrated model combining biomarkers with clinical variables achieved the highest discrimination (AUC = 0.876, 95% CI: 0.841–0.911). The reference diagonal represents a model with no discriminatory ability (AUC = 0.5).

ROC curve analysis identified optimal biomarker cut-offs: CRP >10 mg/L (sensitivity 65.2%, specificity 72.6%, AUC = 0.728, 95% CI: 0.681–0.775); PCT >0.05 ng/mL (sensitivity 54.3%, specificity 76.6%, AUC = 0.701, 95% CI: 0.651–0.751); MAU ≥30 mg/24 h (sensitivity 55.4%, specificity 73.4%, AUC = 0.682, 95% CI: 0.630–0.734). Patients with elevated biomarkers had substantially higher CIN rates: CRP >10 mg/L, 35.7% vs. 11.7% (*p* < 0.001); PCT >0.05 ng/mL, 35.2% vs. 10.5% (*p* < 0.001); MAU ≥30 mg/24 h, 32.7% vs. 11.6% (*p* < 0.001). Among patients with all three biomarkers elevated (*n* = 58), CIN incidence reached 50.0%, compared with 5.8% in those with all three normal (*n* = 206, *p* < 0.001). Table 2 presents comprehensive performance metrics for individual biomarkers and the combined panel. The ROC curves for all individual biomarkers, the combined three-biomarker panel, and the integrated model are illustrated in Figure 1, visually demonstrating the incremental gain in discriminatory performance achieved by each successive combination strategy.

**Table 2:** Discriminatory performance of individual biomarkers and combined panel.

Biomarker	Cutoff	Sensitivity (%)	Specificity (%)	PPV (%)	NPV (%)	AUC (95% CI)
CRP (mg/L)	>10	65.2	72.6	35.7	89.3	0.728 (0.681–0.775)
PCT (ng/mL)	>0.05	54.3	76.6	35.2	88.1	0.701 (0.651–0.751)
MAU (mg/24 h)	≥30	55.4	73.4	32.7	87.9	0.682 (0.630–0.734)
Combined score ≥2	—	56.5	80.7	40.6	88.8	0.856 (0.818–0.894)

PPV, positive predictive value; NPV, negative predictive value; AUC, area under the ROC curve; CI, confidence interval.

Combined score refers to the number of elevated biomarkers (0–3). Cutoffs determined by maximizing the Youden index.

### 3.6 Multivariable predictors of CIN

**Figure 2:**
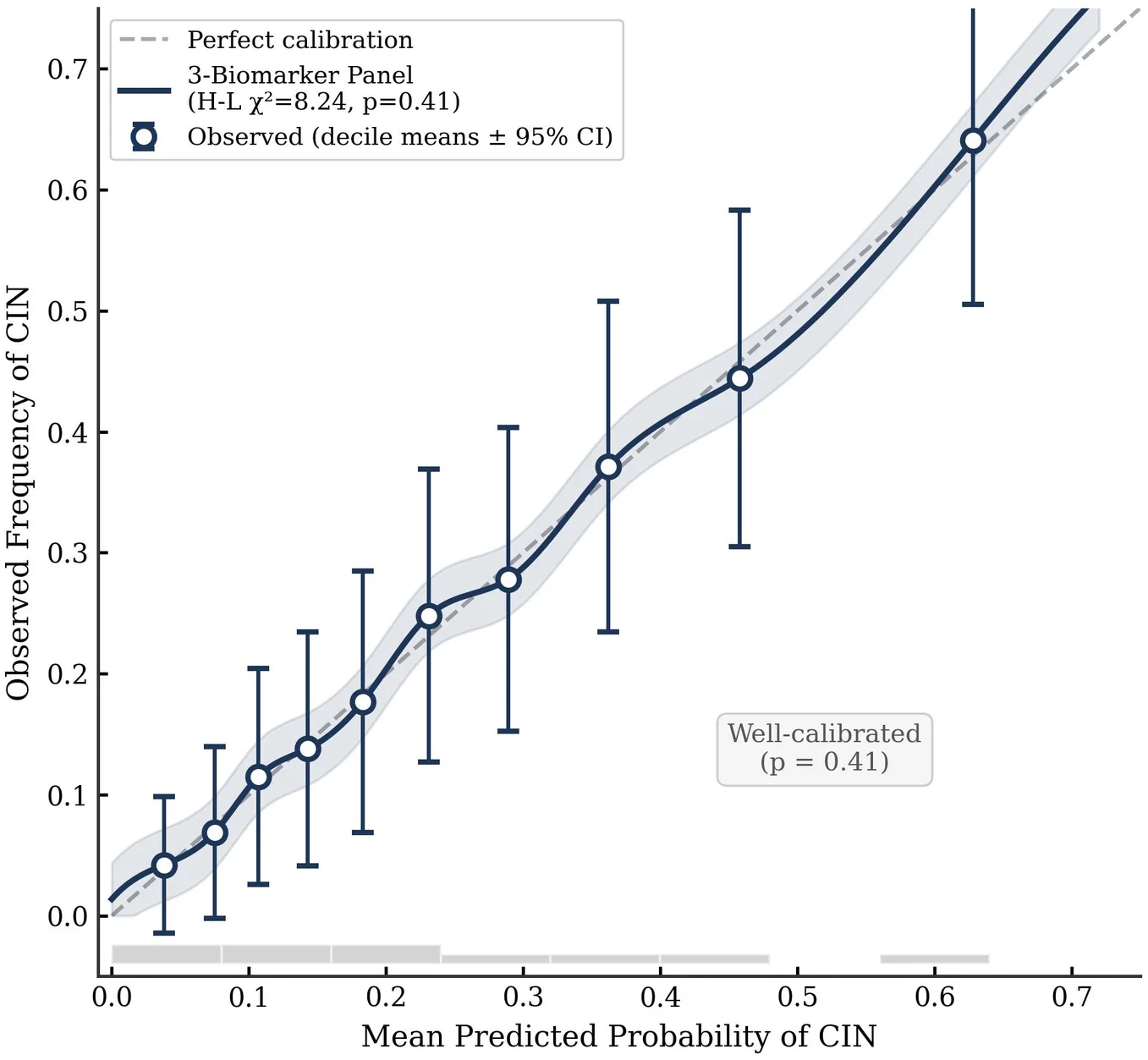
Calibration plot for the three-biomarker panel model. The calibration curve (solid line) shows excellent agreement between predicted and observed CIN probabilities across all deciles of predicted risk (Hosmer-Lemeshow *χ*^2^ = 8.24, *p* = 0.41). Data points represent decile-mean observed frequencies (○) with 95% confidence interval error bars. The shaded region denotes the 95% confidence band of the calibration curve. The dashed diagonal represents perfect calibration. Minimal deviation from the ideal line across the full spectrum of predicted probabilities confirms the model is well-calibrated and suitable for individual-level risk communication in cardio-oncology practice.

Table 3 presents univariable and multivariable logistic regression results. In multivariable analysis, independent CIN predictors included: CRP >10 mg/L (OR = 3.15, 95% CI: 1.82–5.45, *p* < 0.001), PCT >0.05 ng/mL (OR = 2.87, 95% CI: 1.65–4.99, *p* < 0.001), MAU ≥30 mg/24 h (OR = 3.42, 95% CI: 2.01–5.82, *p* < 0.001), diabetes mellitus (OR = 2.18, 95% CI: 1.35–3.52, *p* = 0.002), contrast volume >200 mL (OR = 2.45, 95% CI: 1.48–4.06, *p* < 0.001), baseline eGFR <60 mL/min/1.73 m^2^ (OR = 4.28, 95% CI: 2.52–7.27, *p* < 0.001), heart failure (OR = 1.92, 95% CI: 1.02–3.63, *p* = 0.044), and age >70 years (OR = 1.75, 95% CI: 1.05–2.92, *p* = 0.032). The full model demonstrated excellent discrimination (AUC = 0.876, 95% CI: 0.841–0.911) and good calibration across all deciles of predicted risk (Hosmer-Lemeshow *χ*^2^ = 8.24, *p* = 0.41), with graphical calibration assessment illustrated in Figure 2, confirming close agreement between model-predicted and observed CIN probabilities.

**Table 3:** Univariable and multivariable logistic regression for CIN prediction.

Variable	Univariable OR (95% CI)	*P*-value	Multivariable OR (95% CI)	*P*-value
Age >70 years	2.08 (1.32–3.28)	0.002	1.75 (1.05–2.92)	0.032
Female sex	1.13 (0.70–1.83)	0.609	—	—
Diabetes mellitus	2.52 (1.61–3.94)	<0.001	2.18 (1.35–3.52)	0.002
Heart failure	2.31 (1.26–4.24)	0.007	1.92 (1.02–3.63)	0.044
Baseline eGFR <60	5.28 (3.28–8.52)	<0.001	4.28 (2.52–7.27)	<0.001
LVEF <50%	2.15 (1.28–3.62)	0.004	1.48 (0.82–2.68)	0.193
CRP >10 mg/L	4.95 (3.08–7.95)	<0.001	3.15 (1.82–5.45)	<0.001
PCT >0.05 ng/mL	3.85 (2.42–6.14)	<0.001	2.87 (1.65–4.99)	<0.001
MAU ≥30 mg/24 h	3.42 (2.15–5.44)	<0.001	3.42 (2.01–5.82)	<0.001
Contrast vol >200 mL	4.18 (2.65–6.59)	<0.001	2.45 (1.48–4.06)	<0.001
CV/CrCl ratio >3	3.92 (2.48–6.18)	<0.001	1.56 (0.88–2.78)	0.129
Mehran score ≥11	4.52 (2.85–7.17)	<0.001	—	—

Multivariable model includes variables with *p* < 0.10 in univariable analysis using backward stepwise selection.

OR, odds ratio; CI, confidence interval; NS, not significant; eGFR, estimated glomerular filtration rate; LVEF, left ventricular ejection fraction; CRP, C-reactive protein; PCT, procalcitonin; MAU, microalbuminuria; CV/CrCl, contrast volume-to-creatinine clearance ratio.

### 3.7 Combined biomarker panel performance

An additive biomarker score was constructed by assigning one point for each elevated marker (CRP >10 mg/L, PCT >0.05 ng/mL, MAU ≥30 mg/24 h; range 0–3). CIN rates increased dramatically with increasing score: score 0 (*n* = 206): 5.8%; score 1 (*n* = 152): 18.4%; score 2 (*n* = 70): 32.9%; score 3 (*n* = 58): 50.0% (*p* for trend <0.001). The combined three-biomarker panel demonstrated superior discrimination (AUC = 0.856, 95% CI: 0.818–0.894) compared to all individual markers (*p* < 0.001 for all comparisons). Adding clinical variables further improved discrimination to AUC = 0.876 (95% CI: 0.841–0.911, *p* = 0.042 vs. biomarkers alone). Bootstrap resampling (1,000 iterations with full model re-derivation) yielded an optimism-corrected AUC of 0.841. The biomarker panel correctly reclassified 28.3% of patients compared to clinical variables alone (NRI = 0.283, *p* < 0.001), with an IDI of 0.142 (*p* < 0.001). Table 4 presents detailed model comparison metrics. A comprehensive visual comparison of ROC curves across all models is provided in Figure 1, clearly demonstrating the stepwise improvement in discriminatory performance achieved by the combined biomarker approach. The excellent calibration of the combined model is further confirmed in Figure 2, with the calibration curve closely following the ideal 45-degree line across the full spectrum of predicted CIN probabilities.

**Table 4:** Comparative discriminatory performance of biomarker and clinical models.

Model	AUC (95% CI)	Sensitivity (%)	Specificity (%)	NRI (*p*-value)	IDI (*p*-value)
Mehran score	0.714 (0.665–0.763)	58.7	75.4	—	—
CRP alone	0.728 (0.681–0.775)	65.2	72.6	—	—
PCT alone	0.701 (0.651–0.751)	54.3	76.6	—	—
MAU alone	0.682 (0.630–0.734)	55.4	73.4	—	—
3-biomarker panel	0.856 (0.818–0.894)	71.7	81.2	0.283 (<0.001)	0.142 (<0.001)
Clinical variables	0.832 (0.792–0.872)	69.6	79.2	—	—
Biomarkers + clinical	0.876 (0.841–0.911)	75.0	83.5	0.312 (<0.001)	0.178 (<0.001)

AUC, area under the ROC curve; NRI, net reclassification improvement; IDI, integrated discrimination improvement.

NRI and IDI calculated relative to the Mehran score as reference. The 3-biomarker panel consists of CRP, PCT, and MAU. Clinical variables include age >70 years, diabetes, eGFR <60 mL/min/1.73 m^2^, heart failure, and contrast volume >200 mL.

### 3.8 Biomarker score risk stratification

**Figure 3:**
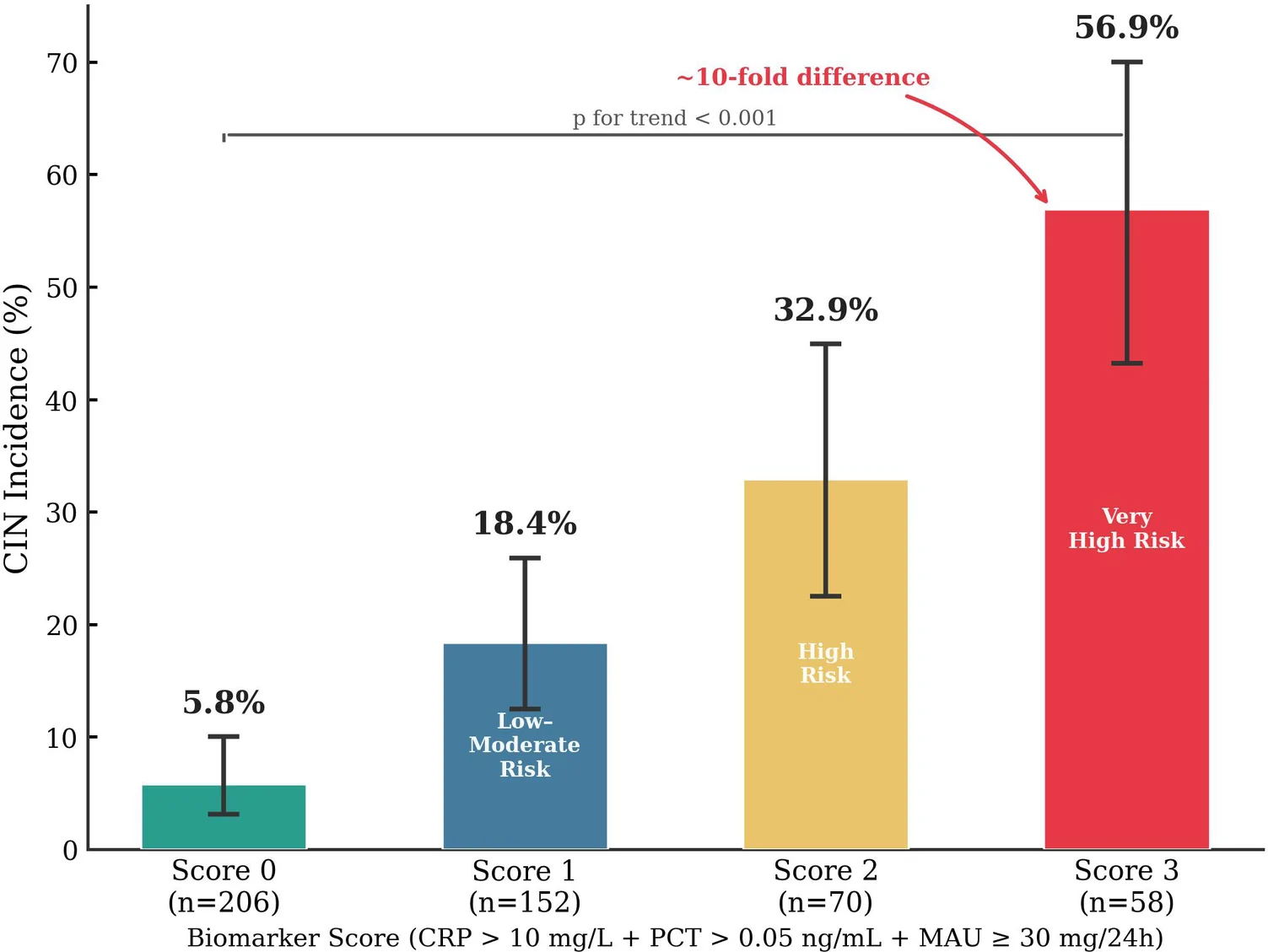
CIN incidence stratified by the additive biomarker score (0–3), based on the number of elevated markers: CRP >10 mg/L, PCT >0.05 ng/mL, and MAU ≥30 mg/24 h. CIN rates were 5.8% (score 0), 18.4% (score 1), 32.9% (score 2), and 50.0% (score 3), representing an approximately 8.6-fold difference between the lowest- and highest-risk groups (*p* for trend <0.001). Error bars denote 95% confidence intervals. Bar colors indicate risk category: very low (score 0), low–moderate (score 1), high (score 2), and very high (score 3). This figure visually emphasizes the clinically meaningful, step-wise risk stratification provided by the simple additive biomarker score, directly supporting risk-adapted preventive decision-making in cardio-oncology practice.

Table 5 presents detailed CIN risk stratification by biomarker score. The score demonstrated an excellent gradient in CIN risk with clear clinical separation between groups. Patients with score 0 had a very low CIN risk (5.8%), while those with score 3 had an extremely high risk (50.0%), representing an approximately8.6-fold risk difference—a gradient that provides meaningful clinical decision support for pre-procedural risk assessment. As illustrated in Figure 3, the step-wise escalation in CIN incidence across biomarker score categories was both statistically robust (*p* for trend <0.001) and visually striking, with non-overlapping 95% confidence intervals between the lowest- and highest-risk groups, underscoring the practical utility of this simple scoring system for individualized risk communication and preventive strategy selection.

**Table 5:** CIN risk stratification by additive biomarker score.

Biomarker score	*N* (%)	CIN *n* (%)	Severe CIN *n* (%)	OR (95% CI)	Risk category
0	206 (42.4%)	12 (5.8%)	0 (0%)	1.00 (reference)	Very low
1	152 (31.3%)	28 (18.4%)	2 (1.3%)	3.64 (1.81–7.32)	Low-moderate
2	70 (14.4%)	23 (32.9%)	2 (2.9%)	7.95 (3.78–16.71)	High
3	58 (11.9%)	29 (50.0%)	3 (5.2%)	16.17 (7.39–35.38)	Very high
*P* for trend	—	<0.001	0.021	<0.001	—

Biomarker score ranges from 0 to 3 based on the number of elevated markers (CRP >10 mg/L, PCT >0.05 ng/mL, MAU ≥30 mg/24 h). Severe CIN defined as requiring renal replacement therapy. OR = odds ratio calculated relative to score 0.

### 3.9 Clinical outcomes and hospital course

**Figure 4:**
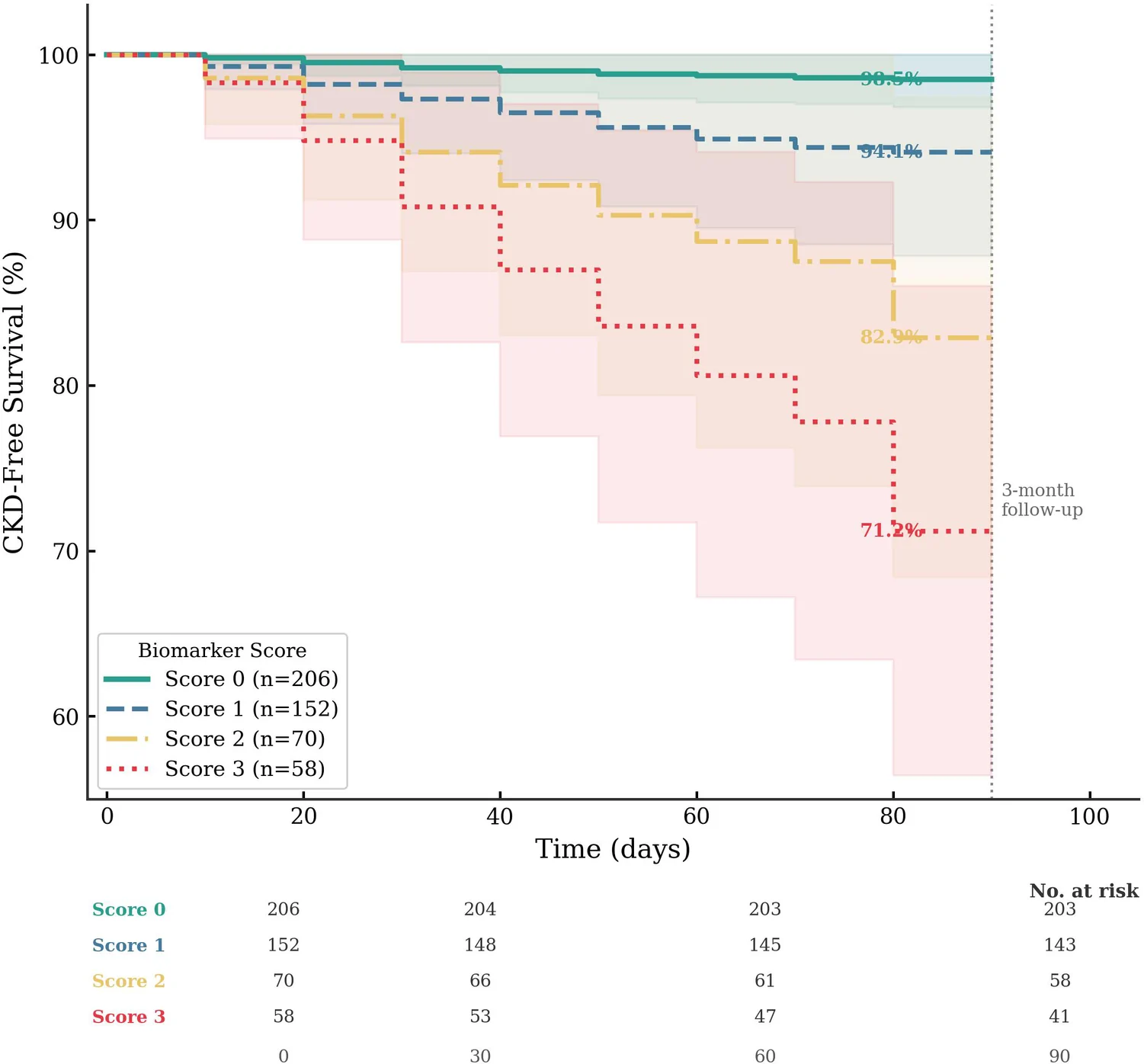
Kaplan–meier curves for chronic kidney disease (CKD)-free survival at 3-month follow-up, stratified by biomarker score. Patients with higher biomarker scores demonstrated significantly worse renal outcomes, with clear and consistent separation of survival curves across the 90-day follow-up period (log-rank *p* < 0.001). CKD-free survival rates at 3 months were: score 0 (98.5%), score 1 (94.1%), score 2 (82.9%), and score 3 (71.2%). Shaded regions represent 95% confidence bands. The values 0, 30, 60, and 90 denote follow-up time points in days and appear solely as the *x*-axis labels; the numbers-at-risk table beneath the axis lists, for each of the four biomarker-score strata, the count of patients still at risk at each time point. Because patients are removed from the at-risk set once they die or reach the CKD endpoint, the total at-risk count at day 90 (445) is necessarily lower than the 478 patients with available 3-month follow-up: the 33-patient difference comprises 11 deaths and 22 CKD events occurring before day 90, while the remaining 14 CKD events were ascertained at the completed 3-month assessment, reconciling with the 36 CKD events (12 non-CIN, 24 CIN) reported in Table 6. These findings illustrate that the prognostic value of the biomarker panel extends beyond prediction of acute CIN to encompass longer-term renal outcomes with direct relevance for post-procedural anticancer treatment planning in cancer survivors.

Patients developing CIN experienced significantly worse clinical outcomes. Median hospital stay was 7.0 days (IQR: 5.0–10.0) in CIN patients vs. 3.0 days (IQR: 2.0–4.0) in non-CIN patients (*p* < 0.001). In-hospital mortality was 4.3% (4/92) vs. 0.5% (2/394) (*p* = 0.005). At 3-month follow-up (completed in 478 patients, 98.4%), chronic kidney disease—defined as sustained eGFR decline ≥25% from baseline—occurred in 27.3% of CIN patients vs. 3.1% of non-CIN patients (*p* < 0.001). The combined endpoint of death or dialysis at 3 months occurred in 8.0% of CIN patients vs. 0.5% of non-CIN patients (OR = 16.5, 95% CI: 3.4–80.2, *p* < 0.001). Table 6 presents detailed clinical outcome data. Kaplan–Meier analysis further demonstrated that the prognostic impact of CIN extended across the biomarker score strata, with progressively worse 3-month CKD-free survival in higher-score groups (Figure 4; log-rank *p* < 0.001): CKD-free survival at 3 months was 98.5% for score 0, declining to 94.1%, 82.9%, and 71.2% for scores 1, 2, and 3, respectively—indicating that the biomarker panel's predictive value extends beyond the acute procedural period to inform longer-term renal prognosis with direct relevance for post-procedural anticancer treatment planning.

**Table 6:** Clinical outcomes stratified by CIN Status.

Outcome	No CIN (*n* = 394)	CIN (*n* = 92)	*P*-value
Peak Cr increase (mg/dL)	0.08 ± 0.12	0.78 ± 0.32	<0.001
Time to peak Cr (hours)	—	48 (36–60)	—
Length of stay (days)	3.0 (2.0–4.0)	7.0 (5.0–10.0)	<0.001
ICU admission	18 (4.6%)	22 (23.9%)	<0.001
Severe CIN (dialysis)	0 (0%)	7 (7.6%)	<0.001
In-hospital mortality	2 (0.5%)	4 (4.3%)	0.005
30-day mortality	5 (1.3%)	6 (6.5%)	0.003
Chronic kidney disease (3-mo)	12/390 (3.1%)	24/88 (27.3%)	<0.001
Dialysis dependency (3-mo)	0/390 (0%)	3/88 (3.4%)	0.001

Values are mean ± SD, median (IQR), or *n* (%).

Cr, creatinine; ICU, intensive care unit.

Severe CIN defined as requiring renal replacement therapy during index hospitalization. Chronic kidney disease defined as sustained eGFR decline ≥25% from baseline at 3-month follow-up.

## 4 Discussion

This retrospective cohort study demonstrates that combined pre-procedural assessment of CRP, PCT, and MAU provides excellent discrimination (AUC = 0.856) for predicting contrast-induced nephropathy following PCI, substantially superior to individual biomarkers and the traditional Mehran score. Because the cohort excluded patients on active anticancer therapy and included only a minority (11.9%) with a cancer history, the implications for cardio-oncology practice are framed as a mechanistically plausible hypothesis rather than a demonstrated application; in that context, patients undergoing contrast-based cardiac procedures may harbor elevated inflammatory and renal vulnerability profiles attributable to malignancy and anticancer therapy. The simple additive biomarker score—assigning one point for each elevated marker—demonstrated strong risk stratification with an approximately 8.6-fold gradient in CIN incidence (5.8% to 50.0%), providing a clinically practical framework for evidence-based, personalized preventive strategies.

The 18.9% CIN incidence observed in our cohort—comprising patients with substantial cardiometabolic comorbidity burden including 34.6% with diabetes, 11.5% with heart failure, and 33.3% with acute coronary syndrome presentations—provides relevant context for cancer cardiology populations. Cancer patients undergoing contrast-based cardiac procedures frequently present with similar or greater cardiometabolic risk burdens, further amplified by treatment-related renal injury, systemic inflammation, and endothelial dysfunction. Epidemiological evidence suggests CIN rates exceeding 25%–30% in cancer patients receiving nephrotoxic chemotherapy (particularly platinum-based regimens) who subsequently require iodinated contrast procedures (10). The biomarker panel investigated in the present study could provide particularly strong clinical value in stratifying ultra-high-risk individuals within this vulnerable cancer cardiology population, enabling targeted deployment of intensive preventive measures.

CRP emerged as a robust independent predictor of CIN (OR = 3.15 for >10 mg/L), consistent with mounting evidence implicating systemic inflammation in contrast nephrotoxicity susceptibility. In cancer cardiology contexts, CRP elevation reflects not only pre-existing cardiovascular inflammation but also malignancy-associated systemic inflammatory states, cytokine release from immune checkpoint inhibitors, and subclinical endothelial injury from anthracyclines and antiangiogenic agents. These mechanisms impair nitric oxide bioavailability, heighten oxidative stress, activate complement pathways, and promote procoagulant activity—all pathophysiologic processes potentiating CIN. The convergence of cancer treatment-related inflammation with contrast-mediated tubular toxicity creates a “two-hit” model of acute renal injury that CRP monitoring could efficiently identify prior to contrast-based cardiac procedures. Importantly, the >10 mg/L threshold corresponds to a clinically actionable CRP level routinely measured in cardio-oncology surveillance panels.

Procalcitonin demonstrated independent predictive value (OR = 2.87 for >0.05 ng/mL). We emphasize that this cut-off was data-driven (derived by maximizing the Youden index) rather than pre-specified, and should be regarded as exploratory pending external confirmation. In this general PCI cohort, the marker is most plausibly interpreted as an index of low-grade systemic inflammatory stress; we therefore no longer ascribe its predictive value to immunotherapy-related mechanisms such as CAR-T or checkpoint-inhibitor toxicity, which were not represented in this population. Because CRP and PCT were moderately correlated (r = 0.58), we formally quantified the incremental contribution of PCT conditional on CRP status: adding PCT to a CRP-containing model yielded a category-free NRI of 0.118 (*p* = 0.02) and an IDI of 0.041 (*p* = 0.03), indicating modest but statistically significant additional discrimination. Even below traditional sepsis thresholds (>0.5 ng/mL), mild PCT elevations at the >0.05 ng/mL threshold indicate heightened microvascular inflammatory stress that may amplify renal vulnerability to contrast injury, and PCT's rapid kinetics may capture acute inflammatory cascades missed by CRP. Whether PCT monitoring offers comparable value in cancer patients on immunotherapy or cellular therapies remains a hypothesis requiring dedicated study in cardio-oncology cohorts.

Microalbuminuria proved the strongest individual predictor of CIN (OR = 3.42 for ≥30 mg/24 h), underscoring the critical importance of baseline renal endothelial integrity in determining contrast nephrotoxicity susceptibility. In cancer patients, MAU serves as an integrative biomarker capturing the cumulative renal impact of nephrotoxic chemotherapy, antiangiogenic agents that disrupt VEGF-mediated glomerular permselectivity, radiation nephropathy, and treatment-related hypertension. The 32.1% MAU prevalence despite a mean eGFR of 78.5 mL/min/1.73 m^2^ in our cohort illustrates a critical clinical phenomenon: conventional kidney function metrics substantially underestimate true renal vulnerability—a discordance even more pronounced in cancer patients where GFR may remain preserved despite significant nephron loss from prior platinum-based or other nephrotoxic treatments. MAU monitoring thus provides a sensitive indicator of subclinical renal damage and reduced nephron reserve that creatinine-based assessments systematically fail to detect. From a cancer cardiology perspective, MAU also reflects the cumulative cardiovascular toxicity of anticancer treatments through its mechanistic association with systemic endothelial dysfunction, providing additional prognostic information beyond CIN prediction.

The synergistic discriminatory value of combining all three biomarkers (AUC = 0.856) represents the central finding of this study, with direct applications in cancer cardiology risk stratification. The multi-dimensional biomarker approach—simultaneously capturing systemic inflammation (CRP), acute inflammatory stress (PCT), and cumulative renal endothelial damage (MAU)—aligns with the 2022 ESC Guidelines on Cardio-Oncology's emphasis on integrating biomarkers, imaging, and genomic data to establish individualized risk assessment models. For cancer cardiology patients with biomarker score 2–3, the panel supports aggressive preventive strategies including intensive intravenous hydration, contrast volume minimization, consideration of alternative non-contrast cardiac imaging modalities, staged procedures where feasible, post-procedural hemofiltration in highest-risk patients, and intensified monitoring for renal function deterioration (26–31). For patients with biomarker score 0–1, the excellent negative predictive value (94.2% for score 0) provides evidence-based reassurance for proceeding with necessary cardiac procedures under standard precautions without delaying oncological care.

Clinical integration of this biomarker panel into cardio-oncology practice offers additional strategic dimensions relevant to optimizing the balance between tumor treatment efficacy and cardiovascular safety. For cancer patients with elevated biomarker scores requiring elective cardiac catheterization, temporary suspension of nephrotoxic chemotherapy agents, careful scheduling relative to chemotherapy cycles, optimization of hydration status, and management of modifiable risk factors (hyperglycemia, blood pressure, nephrotoxic medications) before procedures could substantially reduce CIN risk without compromising oncological outcomes. The biomarker panel could also facilitate timely communication between cardiologists and oncologists regarding procedural timing and risk, supporting the multidisciplinary collaboration that is foundational to cardio-oncology practice. Furthermore, the panel's prognostic extension to 3-month CKD-free survival outcomes (Figure 4) suggests that biomarker-based risk stratification captures clinically meaningful renal prognosis information relevant to long-term anticancer treatment planning in cancer survivors.

Several limitations warrant acknowledgment. First, the single-center retrospective design may limit generalizability to diverse practice settings and populations. Second, the observational design precludes definitive causal inferences. Although bootstrap internal validation indicated only modest optimism (apparent AUC 0.856 vs. optimism-corrected 0.841), a single-center model remains susceptible to overfitting to the local case-mix, and this gap likely underestimates the true optimism that would be revealed in external data. The relatively low Mehran score AUC observed here (0.714) compared with some published cohorts (∼0.74) further suggests differences in patient selection and case-mix that may not transfer to other settings (32–36). External, ideally multicenter, validation using independent retrospective or prospective cohorts is therefore essential before any clinical implementation, and the data-driven biomarker thresholds should be re-derived and confirmed in independent datasets. Third, while the study population reflects patients with significant comorbidity burdens, it did not specifically enrich for cancer patients actively receiving anticancer therapy; dedicated validation in cancer cardiology cohorts—particularly those receiving immunotherapy, targeted therapies, and cellular therapies—is essential, whether in retrospective registries with complete treatment records or in future prospective studies. Fourth, 24 h urine collection for MAU is more cumbersome in acutely ill oncology patients; validation of spot urine albumin-creatinine ratios as practical surrogates warrants investigation. Fifth, for urgent and acute coronary syndrome presentations, 24 h urine collection was initiated at admission but continued peri-procedurally, introducing a risk of temporal leakage whereby some post-contrast urine may have been included in the MAU measurement; this limits causal interpretation and could introduce reverse causation bias. Future studies, whether retrospective with protocol-driven collection or prospective, should confirm that MAU collection is completed prior to contrast administration in all patients, or validate the spot urine albumin-to-creatinine ratio as a practical surrogate. Sixth, the study was not powered to evaluate outcomes specifically in severe CIN requiring dialysis. Future research should enroll cancer patients on active anticancer therapy in dedicated prospective or registry studies, evaluate biomarker thresholds in treatment-related inflammatory contexts, and determine whether biomarker-guided preventive strategies improve clinical outcomes in randomized cancer cardiology trials.

## 5 Conclusions

Integration of this three-biomarker panel into standard pre-procedural cancer cardiology assessment could enable risk-stratified personalized preventive strategies, inform optimal timing of cardiac procedures relative to anticancer treatment cycles, guide post-procedural renal monitoring protocols, and facilitate multidisciplinary communication between oncologists and cardiologists regarding procedural risk in the context of ongoing cancer management. Future research should validate this biomarker framework in dedicated cancer cardiology cohorts and evaluate whether biomarker-guided preventive interventions reduce CIN incidence and improve renal and oncological outcomes in randomized controlled trials designed for cancer cardiology populations.

## Data Availability

The datasets presented in this study can be found in online repositories. The names of the repository/repositories and accession number(s) can be found below: https://figshare.com/s/25acbcb5247ba6663a86.
